# The Impact of Surgical Experience in VATS Lobectomy on Conversion and Patient Quality of Life: Results from a Comprehensive National Video-Assisted Thoracic Surgical Database

**DOI:** 10.3390/cancers15020410

**Published:** 2023-01-08

**Authors:** Luca Bertolaccini, Giulia Fornaro, Oriana Ciani, Elena Prisciandaro, Roberto Crisci, Rosanna Tarricone, Lorenzo Spaggiari

**Affiliations:** 1 Department of Thoracic Surgery, IEO, European Institute of Oncology IRCCS, 20141 Milan, Italy; 2 Centre for Research on Health and Social Care Management (CERGAS), SDA Bocconi School of Management, 20136 Milan, Italy; 3 Department of Life, Health and Environmental Sciences, Thoracic Surgery Unit, University of L’Aquila, 64100 Teramo, Italy; 4 Department of Oncology and Hemato-Oncology, University of Milan, 20122 Milan, Italy

**Keywords:** lung cancer, VATS lobectomy, seniority, surgical experience, quality of life

## Abstract

**Simple Summary:**

Although unexpected conversion during Video-Assisted Thoracic Surgery (VATS) lobectomy is up to 23%, the effects on postoperative outcomes remain debatable. This retrospective study aimed: (i) to identify potential preoperative risk factors of VATS conversion to standard thoracotomy; (ii) to assess the impact of surgical experience in VATS lobectomy on conversion rate and patient health-related quality of life. Clinical nodal involvement was confirmed as the most critical predictor of conversion. Greater experience in VATS lobectomy did not decrease conversion rate and postoperative complications but was positively associated with postoperative patient quality of life.

**Abstract:**

Objectives: Although unexpected conversion during Video-Assisted Thoracic Surgery (VATS) lobectomy is up to 23%, the effects on postoperative outcomes remain debatable. This retrospective study aimed: (i) to identify potential preoperative risk factors of VATS conversion to standard thoracotomy; (ii) to assess the impact of surgical experience in VATS lobectomy on conversion rate and patient health-related quality of life. Methods: We extracted detailed information on VATS lobectomy procedures performed consecutively (2014–2019). Predictors of conversion were assessed with univariable and multivariable logistic regressions. To assess the impact of VATS lobectomy experience, observations were divided according to surgeons’ experiences with VATS lobectomy. The impact of VATS lobectomy experience on conversion and occurrence of postoperative complications was evaluated using logistic regressions. The impact of VATS lobectomy experience on EuroQoL-5D (EQ-5D) scores at discharge was assessed using Tobit regressions. Results: A total of 11,772 patients underwent planned VATS for non-small-cell lung cancer (NSCLC), with 1074 (9.1%) requiring conversion to thoracotomy. The independent predictors at multivariable analysis were: FEV1% (OR = 0.99; 95% CI: 0.98–0.99, *p* = 0.007), clinical nodal involvement (OR = 1.43; 95% CI: 1.08–1.90, *p* = 0.014). Experienced surgeons performed 4079 (34.7%) interventions. Experience in VATS lobectomy did not show a relevant impact on the risk of open surgery conversion (*p* = 0.13) and postoperative complications (*p* = 0.10), whereas it showed a significant positive impact (*p* = 0.012) on EQ-5D scores at discharge. Conclusions: Clinical nodal involvement was confirmed as the most critical predictor of conversion. Greater experience in VATS lobectomy did not decrease conversion rate and postoperative complications but was positively associated with postoperative patient quality of life.

## 1. Introduction

The importance of Video-Assisted Thoracic Surgery (VATS) lobectomy in the treatment of lung cancer has been intensively studied for more than a decade [1]. VATS lobectomy is associated with decreased morbidity, improved quality of life, and shorter hospital stays, and survival rates are comparable to those for patients who undergo thoracotomic lobectomy [2]. Nowadays, VATS lobectomy is regarded as the standard surgical procedure for early-stage non-small-cell lung cancer (NSCLC), and it has been progressively applied to more advanced lung cancer cases.

The adoption of this procedure by the thoracic surgeons’ community has been sluggish over the past quarter-century for several reasons, including the concern of severe intraoperative complications that have been proven to be rare but potentially catastrophic. Injuries to vascular structures may be irreversible and demand an emergency and unscheduled conversion or, worse, an extensive lung resection [3]. The existing literature lacks a comprehensive checklist of preoperative and intraoperative signals that would warn surgeons of potentially dangerous situations. Increasing the understanding of significant risk factors or near-miss scenarios found during the process may be one of the measures to prevent their occurrence and allow more extensive and safer adoption of the technique [4].

This retrospective intention-to-treat analysis aimed: (i) to identify potential preoperative risk factors of VATS conversion to standard thoracotomy; (ii) to assess the impact of surgeons’ experience in VATS lobectomy on conversion rate and patient health-related quality of life.

## 2. Material and Methods

The Italian VATS Group from the Italian Society of Thoracic Surgery created the Italian VATS lobectomy database in 2013 to collect prospective data on VATS lobectomies. At the time of the data extraction, there were more than 50 participating centres (general thoracic surgery units or services, not individual surgeons).

### 2.1. Ethical Considerations

According to the International Conference on Harmonization Guidelines for Good Clinical Practice, the data were collected anonymously.

An ethical committee approved the registry; the registry presented a clear opt-out option (81/2014/O/Oss). The Institutional Review Board, informed of the database extraction, did not require approval because of the study’s retrospective nature.

This manuscript was written according to the Strengthening the Reporting of Cohort Studies in Surgery (STROCSS) Statement [5]. The STROCSS checklist is available as Supplemental File S1.

### 2.2. Evaluation Outcomes

The primary endpoint was the identification of potential preoperative risk factors in VATS conversion to standard thoracotomy.

The secondary endpoints were: (i) the assessment of the impact of surgical experience in VATS lobectomy on conversion rate; (ii) the impact of surgical experience on patient health-related quality of life.

### 2.3. Settings and Patient Selection

The inclusion criteria were: (i) VATS lobectomy procedures intended as a primary procedure for pulmonary lobectomy; (ii) performed consecutively between 1 January 2014 and 31 December 2019.

The exclusion criteria were: (i) missing clinical, intraoperative, or postoperative data; (ii) patients who underwent lobectomy for pathologies different from NSCLC. Overall, 347 patients were excluded from the analysis.

To study predictors of conversion to open surgery, observations were divided into two groups: interventions performed and concluded by VATS and interventions requiring conversion to thoracotomy for any reason. The incidence of specific complications and its correlation with unplanned conversion to open surgery was studied as a relevant postoperative outcome. To assess the impact of VATS lobectomy experience, observations were divided into two groups: interventions performed by a surgeon with VATS lobectomy experience >50 procedures and ≤50 procedures, according to data and recommendations previously published in the literature [6,7,8,9].

### 2.4. Clinicopathological Parameters

The data collected from the registry included multiple variables: e.g., gender, age, surgical indication (benign, primary, or metastatic), previous chemotherapy or radiotherapy, Charlson Comorbidity Index [6], Eastern Cooperative Oncology Group Performance Status (ECOG PS) [7], side and surgical procedure performed, number of incisions, number of resected lymph nodes, cause of conversion (e.g., vascular injuries, etc.), and pathological stage (according to the Eighth Edition of the TNM Classification for Lung Cancer, American Joint Committee on Cancer) [8]. Unfortunately, the database lacked more information on the detailed mechanisms for a portion of the “vascular injury” rough record.

### 2.5. Statistical Analysis

Descriptive statistics were used to summarise data regarding demographic and oncological characteristics. Continuous variables were reported as mean value ± standard deviation and compared between groups using unpaired Student’s t-tests assuming equal variance. The normality of data distribution was assessed with the Kolmogorov–Smirnov test. Categorical variables were reported as absolute value and percentage; differences in their distribution between groups were analysed using chi-square tests.

To identify preoperative clinical risk factors of conversion, univariable logistic regression analysis was performed on a set of variables a priori deemed relevant for conversion, including patient demographics, induction treatment, results of pulmonary function tests, nodal involvement, clinical stage, packages per year, previous surgery, and presence of specific comorbidities. Variables exhibiting a *p*-value ≤ 0.10 were included in the multivariable logistic regression. As a robustness check, a further analysis based on multivariable logistic regressions was implemented by computing standard errors corrected for cluster correlation at the healthcare centre level and including healthcare centre dummy variables (while computing conventional standard errors). The incidence of specific complications and their correlation with conversion to open surgery was assessed by implementing chi-square and Spearman tests and estimating a multivariable logistic model.

The impact of VATS lobectomy experience on conversion and occurrence of postoperative complications (according to Clavien–Dindo [10]) was evaluated using logistic regressions.

The impact of VATS lobectomy experience on EuroQoL-5D (EQ-5D) scores [11] at discharge was assessed using Tobit regressions. Similarly, robustness checks were based on accounting for potential cluster correlation at the healthcare centre level.

A *p*-value < 0.05 was considered statistically significant. Statistical analysis was performed using STATA (StataCorp. 2021. Stata Statistical Software: Release 17. College Station, TX, USA: StataCorp LLC).

## 3. Results

**Figure 1:**
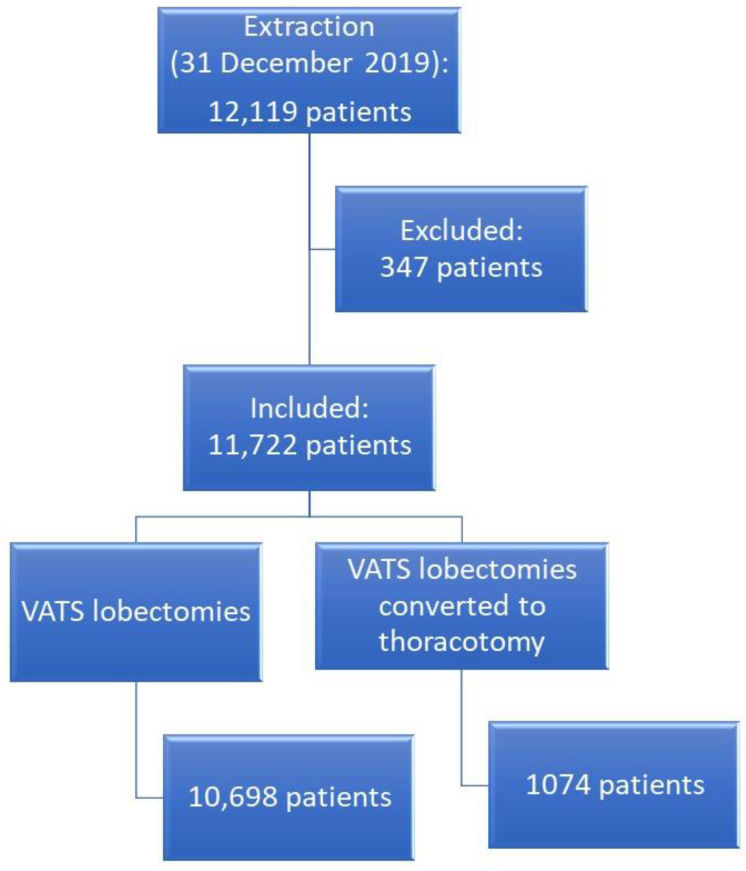
Flow chart of patient selection.

A total of 11,772 cases were included in the analysis (Figure 1).

### 3.1. Risk Factors of Vats Conversion to Thoracotomy

Of the planned VATS lobectomies, 1074 (9.1%) required conversion to open surgery. The most frequent causes of conversion (Table 1) were 298 cases of (27.8%) thoracoscopically uncontrollable bleeding, followed by 248 (23.0%) severely calcified lymph nodes adherent to the pulmonary artery, and 225 (20.1%) pleural adhesions.

The group requiring conversion showed a significant predominance of men (67.1% vs. 59%, *p* < 0.001). Furthermore, the clinical stage (*p* < 0.001) and nodal involvement (*p* < 0.001) were significantly different between the two groups (Table 2). Moreover, the percentage of patients who underwent neoadjuvant treatment before VATS lobectomy was significantly higher in the group that required conversion (4.7% vs. 3.2%, *p* = 0.05). In the conversion group, the pulmonary function tests showed significantly lower values of Forced Expiratory Volume in the first second (FEV1%) (*p* < 0.001) and diffusion lung carbon monoxide/volume alveolar (DLCO/VA%) (*p* = 0.03); the incidence of chronic obstructive pulmonary disease (COPD) was significantly larger in this group (24.8% vs. 21%, *p* = 0.004) (Table 2).

The univariable analysis of the a-priori selected preoperative variables highlighted the following main significant risk factors: male gender, FEV1%, clinical nodal involvement, and clinical stage.

The multivariable logistic model (Table 3) confirmed FEV1% (odds ratio [OR] = 0.99, confidence interval [CI] 95%: 0.99–1.00, *p* = 0.008), clinical nodal involvement (OR = 1.42, CI 95%: 1.07–1.89, *p* = 0.015), and clinical stage (clinical stage I vs. other stages OR = 0.62, CI 95%: 0.50–0.76, *p* < 0.001) as independent risk factors for conversion. These variables remained robust independent risk indicators even when controlling for potential cluster correlation at the healthcare centre level in the multivariable logistic regressions (Supplemental Table S1).

Postoperative complications occurring in the two groups are detailed in Supplemental Table S2. Blood transfusion (ρ = 0.099, *p* < 0.001) showed the largest correlation with conversion, according to the Spearman correlation test (Supplemental Table S3). Atrial fibrillation (OR = 1.59, CI 95%: 1.29–1.98, *p* < 0.001), pneumonia (OR = 1.55, CI 95%: 1.15–2.08, *p* = 0.004), atelectasis (OR = 1.55, CI 95%: 1.06–2.28, *p* = 0.025), blood transfusion (OR = 3.36, CI 95%: 2.49–4.53, *p* < 0.001), and acute renal failure (OR = 2.07, CI 95%: 1.12–3.80, *p* = 0.020) were significantly associated with conversion in multivariable logistic analysis (Table 4).

### 3.2. Impact of Surgical Experience in Vats Lobectomy on Conversion

Regarding surgical experience in VATS lobectomy (Table 2), the two groups had balanced preoperative baseline patient characteristics, except for a significant predominance of men (*p* = 0.005) and smokers (*p* < 0.001) and a lower number of patients who underwent neoadjuvant treatment (*p* < 0.001) in the low-experience group. Multivariable logistic regressions revealed that VATS lobectomy experience was not significantly correlated with conversion or postoperative complications.

### 3.3. Impact of Surgical Experience on Patient Health-Related Quality of Life

Multivariable Tobit regressions (Supplemental Table S4) highlighted a positive, significant correlation of VATS lobectomy experience with the EuroQol-5D score reported by patients at discharge, the effect being robust to model variations in terms of adding control variables (*p* < 0.01) and accounting for potential cluster correlation at the healthcare centre level (*p* < 0.05).

## 4. Discussion

Pulmonary lobectomy remains the gold standard treatment for early-stage NSCLC [1,12,13]. Over the last twenty years, VATS has replaced open thoracotomy as the preferred approach for lobectomy due to its many undeniable advantages: improved perioperative outcomes and patient quality of life, and reduced length of stay and postoperative pain [14,15,16]. Compared with open thoracotomy, VATS ensures similar long-term oncological outcomes in patients who undergo lobectomy for NSCLC while reducing lung cancer-related costs [17]. However, despite being an established procedure, VATS lobectomy carries an inherent risk of intraoperative events (namely vascular injuries) requiring emergency conversion to thoracotomy [4]. Unplanned conversion may also be ascribed to unfavourable anatomy (pleural adhesions, lung emphysema, tissue fibrosis due to previous induction treatments, large tumour size), challenging lymph node dissection (calcifications, sclerosis, bulkiness, infiltration of vascular structures), and/or technical failure of equipment (difficult maintenance of one-lung ventilation) [18,19]. Conversion occurs in up to 23% of VATS procedures; however, several studies have shown an inverse correlation between thoracoscopic surgical experience and the frequency of conversion, with high-volume VATS lobectomy units achieving lower conversion rates [18,19]. Nonetheless, in several European and United States centres, VATS lobectomy is still an underused technique due to the strong concern for intra- and postoperative morbidity associated with the conversion to thoracotomy [20,21,22]. In particular, conversion has been related to an increased rate of pulmonary and/or cardiovascular complications, higher transfusion rate, longer chest-tube-dwelling time, more extended intensive care unit (ICU) stay, and longer hospital length of stay [19,23], although evidence is not unanimous [24].

In our study, we disclosed a significantly higher (*p* < 0.001) postoperative morbidity in the conversion group. Namely, conversion was associated with an increased frequency of cardiovascular events (atrial fibrillation, myocardial infarction, cardiac arrest), respiratory complications (prolonged air leaks, acute respiratory distress syndrome, pneumonia, atelectasis, sputum retention, pleural effusion, emphysema, broncho-pleural fistula, re-intubation and prolonged mechanical ventilation), and other complications (recurrent nerve palsy, acute kidney failure, diarrhoea), as well as blood transfusions and postoperative ICU admission. Similarly, Seitlinger et al. showed a relevant increase in the prevalence of atrial fibrillation and pneumonia after conversion [25]. Tong and colleagues reported a significantly higher frequency of pulmonary complications (including atelectasis and pulmonary infections) among the patients who underwent conversion, whereas the cardiovascular event rate was comparable between the two groups [19]. Gabryel et al. demonstrated that supraventricular arrhythmias, blood transfusions, and postoperative admission to ICU were significantly more common in the conversion group [23]. These findings are consistent with previously published data, disclosing lower cardio-respiratory morbidity after VATS lobectomy, which may be attributed to the limited chest wall trauma (when compared with thoracotomy), resulting in reduced postoperative pain, less impairment of the respiratory mechanics and, consequently, of the pulmonary function [16,26]. Moreover, the greater need for blood transfusion may result from the blood loss caused by possible vascular injuries that require the conversion.

We also aimed to identify potential predictors of conversion to thoracotomy. Our analyses showed that reduced pulmonary function (FEV1%), clinical nodal involvement, tumour stage >I, and smoking status were significant independent risk factors for conversion, even after adjusting for potential cluster correlation at the healthcare centre level. Age, male gender, and induction therapy were not relevant predictive indicators in our population. A recent meta-analysis on the conversion of VATS to thoracotomy for anatomical lung resections identified several risk factors for conversion, including age, male gender, induction therapy, tumour size, and lymph node disease [27]. However, the authors reported a high variability among studies (cut-off values, inclusion criteria, extent of lung resection) which prevented them from performing an actual meta-analysis.

Nevertheless, some general assumptions can be made. First, regardless of surgical experience, conversion seems more likely to occur in large tumours and difficult lymph node dissection (calcifications, adhesions to vessels/bronchi) as the visualisation of the anatomical structures and tissue handling become more challenging. Secondly, preoperative induction treatments (still considered an absolute contraindication to VATS in some centres) and previous ipsilateral thoracic surgery are usually responsible for an intense inflammatory response that generates hilar and pleural adhesions, thus hindering the achievement of fully thoracoscopic lung resections. In this regard, it has been reported that male patients present more frequently with pleural adhesions, which may explain why the male gender is associated with conversion by several authors [18,19,26]. In our study, surgical experience in VATS lobectomy (>50 procedures) did not affect the conversion rate nor complication rate but was positively associated with postoperative patient quality of life. A significantly greater number of VATS lobectomies which followed neoadjuvant treatment was performed by experienced surgeons, reflecting broader criteria in the selection of VATS candidates. In contrast with our findings, Seitlinger and colleagues observed a downward trend in conversion with the increase in the surgeon’s volume, albeit not proven statistically [25]. The concept that lung cancer patients may benefit from the implementation of a minimal volume standard due to the specialisation of high-volume centres has been described in the literature. A recent prospective multicentre study revealed that distance from a surgery department is not associated with having an operation or death, despite claims by other authors that centralisation results in an increasing travel burden for patients and thus creates a barrier for certain segments of the population to access quality care. In addition, future research should investigate whether the healthcare system could benefit from the centralisation of specialised healthcare services, as it is still unknown whether centralisation results in economies of scale and is cost-effective [28]. Prior research has demonstrated that procedure volume alone may not capture crucial care activities that affect postoperative outcomes. The practice types of surgeons with the most and least experience varied significantly. In the early stages of their careers, surgeons were less likely to conduct high-risk surgeries and more likely to perform procedures on children with fewer risk factors. Senior surgeons were more likely to undertake revision procedures and to handle situations of increasing complexity or rarity. These results imply that a surgeon’s practice is dynamic, and those distinctions in case type, complexity, and independence may evolve. Consequently, it may be challenging to account for these variables when evaluating the effect of surgeon age and experience on clinical results. Younger surgeons, for instance, may begin their careers using either a formal or informal apprenticeship model, in which a senior surgeon is accessible to review clinical decision-making or participate in some stages of an operation. In this sense, younger surgeons may be offered a time of protection during their early careers that is unavailable to those later in their professions. It is also feasible that senior surgeons supervise several learners in the operating room or that they are more inclined to delegate the surgery to a junior colleague, fellow, or resident [29]. Tong et al. revealed that lower surgical experience (<500 procedures/year) was an independent risk indicator for conversion [19]. However, as highlighted by Power and colleagues, many confounding factors may interfere when assessing the impact of experience on conversion [27]. There is a high variability concerning VATS techniques (number and position of ports), case selection, and even management strategies for intraoperative adverse events (conversion by default vs. attempts at treating complications by VATS). Indeed, a systematic recourse to conversion (regardless of surgical experience) offers a plausible explanation for our results and is the favoured approach to intraoperative complication management by many surgeons, who strongly advocate for “patient safety first” and oppose the view of conversion as a “failure”. Patient prognosis is not compromised after conversion, and thoracotomy may ensure better control of unexpected adverse events [30,31]. On the other hand, there is certainly a trend towards fully thoracoscopic management of intraoperative complications and an extension of the inclusion criteria for VATS lobectomy (large tumours, chest wall involvement, sleeve resections) [32,33,34]. Moreover, the rising number of significant and complex lung resections performed by robot-assisted thoracic surgery (RATS) prompted some authors to compare the conversion rate of VATS and RATS lobectomies: Servais and colleagues reported a significantly lower incidence of conversion during RATS, especially in centres with low VATS volume [35].

### Limitations

Our research has several limitations. The investigation was hampered by its retrospective nature. The centres did not describe selection criteria for VATS lobectomy, potentially resulting in classification bias. Nevertheless, as previously demonstrated for other significant database changes, the selection criteria may vary between centres [36]. Unfortunately, the exact mechanisms of vascular injuries and their management are not captured in the database; to accurately detect these complications, an intention-to-treat field containing the details of the planned surgical procedure should be included. The absence of this data could result in an underestimation of the total number of injuries (missing denominator). In addition, the timing of vascular damage is unknown. The precise moment of conversion may shed light on intraoperative decision-making and factors influencing the timely anticipation of probable complications. Defining operative experience may follow different approaches, from threshold on several index procedures performed to operative performance evaluation [37]. Our choice to dichotomise surgeons’ experience > or ≤50 procedures may neglect relevant aspects related to experience development, such as the ordering of the procedure for the individual operator. However, this information was not available in our database.

## 5. Conclusions

Vascular injuries during VATS lobectomy represent an event that directly affects the postoperative outcome. Our analyses confirmed clinical nodal involvement as the most critical predictor of conversion. Experience with VATS lobectomy did not reduce the conversion rate or the incidence of postoperative complications, but it was positively correlated with postoperative patient health-related quality of life.

## Figures and Tables

**Table 1:** Causes of conversion to thoracotomy. The total number of conversions is higher than the total number of cases that required conversion because, in some patients, more than one cause determined the conversion (e.g., severely calcified lymph node on pulmonary artery and uncontrollable bleeding).

Cause of Conversion	No.	%
Uncontrollable bleeding	298	27.8
Lymph nodes stuck on pulmonary artery	248	23.1
Pleural adhesions	225	21.0
Incomplete interlobar fissure	169	15.7
Anatomic anomalies	76	7.1
Advanced staging	72	6.7
Difficult localisation of tumour	67	6.2
Tumour crossing fissure	53	4.9
Time limit	15	1.4
Chest wall invasion	9	0.8
Wrong position of incisions	5	0.5

**Table 2:** Comparisons by lobectomy groups (VATS versus converted to thoracotomy) and VATS lobectomy experience of the first operator (>50 VATS versus ≤50 VATS lobectomies). Values are expressed as numbers (percentages) as otherwise defined. BMI = body mass index; COPD = chronic obstructive pulmonary disease; DLCO = diffusing capacity of the Lung for Carbon Monoxide; FEV1 = forced expiratory volume in 1 s; SD = standard deviation.

	Lobectomy			VATS Lobectomy Experience		
Variables	VATS No. = 10,698 (90.9%)	Converted No. = 1074 (9.1%)	*p*-Value	>50 VATS No. = 4079 (64.1%)	≤50 VATS No. = 2284 (35.9%)	*p*-Value
Age, mean ± SD	67.6 ± 9.9	67.9 ± 10.1	0.27	67.7 ± 10.0	68.1 ± 9.4	0.13
Female gender	4.4 (41.0%)	351 (32.7%)	<0.001	1.7 (41.4%)	863 (37.8%)	0.005
BMI, mean ± SD	29.1 ± 32.6	30.2 ± 33.4	0.46	29.2 ± 34.4	29.3 ± 29.4	0.87
Current smoker	4.0 (69.4%)	434 (77.1%)	<0.001	2.8 (67.8%)	1.7 (74.3%)	<0.001
Packages per year, mean ± SD	38.1 ± 39.5	55.0 ± 75.8	<0.001	39.5 ± 86.6	40.1 ± 53.8	0.80
FEV1%, mean ± SD	94.4 ± 20.1	90.2 ± 19.4	<0.001	93.7 ± 18.7	93.8 ± 19.7	0.93
DLCO%, mean ± SD	83.1 ± 21.7	81.4 ± 19.5	0.03	82.7 ± 17.6	83.8 ± 18.0	0.041
Neoadjuvant	338 (3.2%)	51 (4.7%)	0.005	175 (4.3%)	49 (2.1%)	<0.001
Comorbidities						
Cardiovascular disease	1.3 (12.0%)	141 (13.1%)	0.29	499 (12.2%)	289 (12.7%)	0.63
COPD	2.2 (21.0%)	266 (24.8%)	0.004	892 (21.9%)	563 (24.6%)	0.011
Diabetes	1.4 (12.8%)	154 (14.3%)	0.15	547 (13.4%)	316 (13.8%)	0.64
Connective tissue disease	254 (2.4%)	29 (2.7%)	0.51	94 (2.3%)	62 (2.7%)	0.31
Chronic kidney disease	319 (3.0%)	36 (3.4%)	0.50	127 (3.1%)	82 (3.6%)	0.31
Right lung	6.4 (60%)	592 (55.3%)	0.003	2.5 (59.5%)	1.4 (59.0%)	0.61
Lobe						
Lower	3.8 (35.5%)	399 (37.3%)	0.003	1.4 (34.4%)	862 (37.7%)	0.030
Lower and middle	28 (0.3%)	7 (0.7%)		21 (0.5%)	6 (0.3%)	
Middle	828 (7.8%)	58 (5.4%)		301 (7.4%)	173 (7.6%)	
Upper	6.0 (56.1%)	606 (56.6%)		2.3 (56.9%)	1.2 (54.0%)	
Upper and middle	32 (0.3%)	0 (0%)		15 (0.4%)	4 (0.2%)	
Clinical Stage						
In situ	21 (0.2%)	1 (0.1%)	<0.001	15 (0.4%)	7 (0.3%)	0.96
IA	2.8 (25.9%)	243 (22.7%)		340 (8.3%)	188 (8.2%)	
IB	3.2 (29.9%)	273 (25.5%)		1.4 (32.8%)	764 (33.5%)	
IC	1.5 (13.9%)	127 (11.9%)		1.0 (25.1%)	590 (25.8%)	
II	83 (0.8%)	6 (0.6%)		57 (1.4%)	32 (1.4%)	
IIA	1.9 (17.7%)	236 (22.1%)		628 (15.4%)	354 (15.5%)	
IIB	539 (5.1%)	73 (6.8%)		253 (6.2%)	137 (6.0%)	
III	550 (5.2%)	90 (8.4%)		310 (7.6%)	161 (7.0%)	
IV	145 (1.4%)	21 (2%)		99 (2.4%)	46 (2.0%)	
cN						
0	9.7 (91.4%)	900 (84.1%)	<0.001	3.6 (88.8%)	2.0 (89.4%)	0.78
1	364 (3.4%)	74 (6.9%)		174 (4.3%)	101 (4.4%)	
2	520 (4.9%)	87 (8.1%)		245 (6.0%)	129 (5.6%)	
3	37 (0.3%)	9 (0.8%)		20 (0.5%)	8 (0.4%)	
VATS converted to thoracotomy				330 (8.1%)	233 (10.2%)	0.004
Estimated blood loss, mean ± SD	123.2 ± 108.2	322.2 ± 433.6	<0.001	128.5 ± 127.3	150.9 ± 225.6	<0.001
Any postoperative complication	2.8 (26%)	418 (39%)	<0.001	1.036 (25.4%)	630 (27.6%)	0.057
EuroQoL-5D score at discharge, mean ± SD	0.9 ± 0.2	0.7 ± 0.2	<0.001	0.9 ± 0.2	0.8 ± 0.2	0.0006

**Table 3:** Univariable and multivariable analysis for preoperative clinical risk factors of conversion. CI = confidence interval; COPD = chronic obstructive pulmonary disease; DLCO = diffusing capacity of the Lung for Carbon Monoxide; FEV1 = forced expiratory volume in 1 s; OR = odds ratio.

	Univariable Analysis			Multivariable Analysis		
Variable	OR	CI 95%	*p*-Value	OR	CI 95%	*p*-Value
Age ≥ 70	1.13	1.00–1.28	0.055	0.91	0.74–1.12	0.38
Female gender	0.79	0.61–0.80	<0.001	0.90	0.71–1.14	0.38
Packages per year	1.01	1.01–1.02	0.02	1.00	1.00–1.00	0.014
COPD	1.24	1.07–1.44	0.004	1.18	0.94–1.48	0.15
Previous surgery	0.86	0.75–0.99	0.04	0.98	0.78–1.22	0.83
FEV1%	0.99	0.99–0.99	<0.001	0.99	0.99–1.00	0.008
DLCO%	1.00	0.99–1.00	0.021			
Preoperative neoadjuvant	1.53	1.13–2.07	0.006	1.32	0.83–2.10	0.25
Clinical nodal involvement	2.00	1.67–2.38	<0.001	1.42	1.07–1.90	0.015
Clinical stage I	0.65	0.58–0.74	<0.001	0.62	0.50–0.76	<0.001
Right lung	0.83	0.73–0.94	0.003	0.93	0.76–1.14	0.49

**Table 4:** Multivariable logistic regression model of complications associated with unplanned thoracotomy. ARDS = adult respiratory distress syndrome; CI = confidence interval; ICU = intensive care unit; OR = odds ratio.

Variable	OR	CI 95%	*p*-Value
Atrial fibrillation	1.59	1.29–1.98	<0.001
Myocardial ischemia and infarction	1.74	0.67–4.56	0.26
Prolonged air leak	1.26	1.01–1.56	0.037
ARDS	1.61	0.74–3.49	0.23
Persistent pleural space	0.82	0.55–1.21	0.31
Pneumonia	1.55	1.16–2.08	0.004
Mechanical ventilation	1.89	0.91–3.91	0.088
Atelectasis	1.55	1.06–2.28	0.025
Sputum retention	1.35	0.65–1.93	0.098
Recurrent laryngeal nerve palsy/dysphonia	1.88	1.00–3.51	0.049
Blood transfusion	3.36	2.49–4.53	<0.001
Acute renal failure	2.07	1.12–3.80	0.02
Prolonged mechanical ventilation	0.80	0.34–1.84	0.59
Postoperative ICU	1.24	0.92–1.66	0.15

## Data Availability

The data underlying this article will be shared upon reasonable request to the corresponding author.

## Supplementary Materials

The following supporting information can be downloaded at: https://www.mdpi.com/article/10.3390/cancers15020410/s1, Table S1: Analysis of the impact of cluster correlation at the healthcare centre level. Multivariable logistic regression estimates are expressed in odds ratios; Table S2: Complications in detail between non-converted and converted cases; Table S3: Spearman correlation test between conversion and complications in detail; Table S4: Logistic (1–8) and Tobit (9–12) regressions. All models feature standard errors except for models 3, 7, and 11, where standard errors are corrected for cluster correlation at the healthcare centre level; File S1: The STROCSS 2021 Guideline.

## Glossary of Abbreviations

CI	Confidence Interval
COPD	Chronic Obstructive Pulmonary Disease
DLCO/VA	Diffusing Capacity of the Lung for Carbon Monoxide divided by Alveolar Volume
ECOG PS	Eastern Cooperative Oncology Group Performance Status
EQ-5D	EuroQoL-5D
ICU	Intensive Care Unit
FEV1%	Forced Expiratory Volume in the 1st second
NSCLC	Non-Small Cell Lung Cancer
OR	Odds Ratio
STROCCS	Strengthening the Reporting of Cohort Studies in Surgery
VATS	Video-Assisted Thoracic Surgery
