# Supplementary material for: The Impact of Surgical Experience in VATS Lobectomy on Conversion and Patient Quality of Life: Results from a Comprehensive National Video-Assisted Thoracic Surgical Database
Source: Cancers (Basel). 2023 Jan 8;15(2):410. doi: 10.3390/cancers15020410 (PMC9857299; doi:10.3390/cancers15020410)
Supplement: Supplementary file 1 [file cancers-15-00410-s001.zip › Supplementaty Tables S1-S4.pdf]

| | Model 1 | Model 2 | Model 3 |
|---|---|---|---|
| **Observations** | 4,341 | 4,341 | 4,164 |
| **Age ≥70** | 0.91 | 0.91 | 0.87 |
| | (0.097) | (0.087) | (0.096) |
| **Female gender** | 0.90 | 0.90 | 0.91 |
| | (0.11) | (0.14) | (0.12) |
| **Preoperative neoadjuvant** | 1.32 | 1.32 | 1.68** |
| | (0.31) | (0.28) | (0.43) |
| **FEV1 %** | 0.99*** | 0.99** | 0.99* |
| | (0.003) | (0.003) | (0.003) |
| **DLCO %** | 1.00 | 1.00 | 1.00 |
| | (0.003) | (0.004) | (0.003) |
| **Clinical nodal involvement** | 1.42** | 1.42** | 1.71*** |
| | (0.21) | (0.24) | (0.27) |
| **Clinical Stage I** | 0.62*** | 0.62*** | 0.57*** |
| | (0.066) | (0.066) | (0.065) |
| **Packages per year** | 1.00** | 1.00** | 1.00** |
| | (0.001) | (0.001) | (0.001) |
| **Right lung** | 0.93 | 0.93 | 0.91 |
| | (0.097) | (0.12) | (0.099) |
| **Previous cancer-related surgery** | 0.94 | 0.94 | 0.93 |
| | (0.11) | (0.11) | (0.12) |
| **COPD** | 1.18 | 1.12 | 1.19 |
| | (0.14) | (0.17) | (0.16) |
| **Healthcare centre dummy** | NO | NO | YES |
| **Pseudo R2** | 0.023 | 0.023 | 0.098 |

*** p<0.01, ** p<0.05, * p<0.1

Supplemental Table S1. Analysis of the impact of cluster correlation at the healthcare centre level. Multivariable logistic regression estimates are expressed in odds ratios. Dependent variable: 1 = conversion to open surgery, 0 = otherwise. Model 1 does not account for cluster correlation at the

healthcare centre level (i.e., standard errors were computed), model 2 computes standard errors corrected for cluster correlation at the healthcare centre level, and model 3 features standard errors and healthcare centre dummy variables.

| Variable | VATS No. = 10,698 (90.9%) | Converted No. = 1,074 (9.1%) | *p-value* |
|---|---|---|---|
| Major | | | |
| • Prolonged mechanical ventilation | 48 (0.5%) | 13 (1.2%) | 0.001 |
| • Postoperative ICU | 417 (4.0%) | 70 (6.5%) | <0.001 |
| Cardiovascular | | | |
| • Atrial fibrillation | 668 (6.2%) | 118 (11.0%) | <0.001 |
| • Myocardial ischemia and infarction | 21 (0.2%) | 6 (0.6%) | 0.018 |
| • Stroke | 25 (0.2%) | 4 (0.4%) | 0.382 |
| Acute cardiovascular | | | |
| • Acute peripheral arterial thromboembolism | 10 (0.1%) | 2 (0.2%) | 0.364 |
| • Cardiac arrest | 9 (0.1%) | 5 (0.5%) | 0.001 |
| Pulmonary | | | |
| • Prolonged air leak | 858 (8.0%) | 116 (10.8%) | 0.002 |
| • Pulmonary embolism | 14 (0.1%) | 4 (0.4%) | 0.053 |
| • ARDS | 36 (0.3%) | 12 (1.1%) | <0.001 |
| • Persistent pleural space | 301 (2.8%) | 32 (3.0%) | 0.755 |
| • Pneumonia | 317 (3.0%) | 63 (6.0%) | <0.001 |
| • Mechanical ventilation | 36 (0.3%) | 18 (1.7%) | <0.001 |
| • Middle lobe torsion | 9 (0.1%) | 1 (0.1%) | 0.923 |
| • Atelectasis | 186 (1.7%) | 40 (3.7%) | <0.001 |
| • Sputum retention | 241 (2.3%) | 45 (4.2%) | <0.001 |
| Surgical | | | |
| • Haemothorax | 127 (1.2%) | 19 (1.8%) | 0.100 |
| • Bronchopleural fistula | 19 (0.2%) | 6 (0.6%) | 0.010 |
| • Chylothorax | 37 (0.4%) | 2 (0.2%) | 0.385 |
| • Phrenic nerve injury/palsy | 15 (0.1%) | 2 (0.2%) | 0.705 |
| • Recurrent laryngeal nerve palsy/dysphonia | 62 (0.6%) | 13 (1.2%) | 0.013 |
| • Blood transfusion | 175 (0.7%) | 70 (6.5%) | <0.001 |
| Renal | | | |

| | | | |
|---|---|---|---|
| • **Acute renal failure** | 50 (0.5%) | 16 (1.5%) | <0.001 |
| • **Urinary tract infection** | 62 (0.6%) | 4 (0.4%) | 0.386 |
| • **Dialysis** | 8 (0.1%) | 1 (0.1%) | 0.836 |
| **Gastrointestinal** | | | |
| • **Diarrhoea** | 24 (0.2%) | 7 (0.7%) | 0.009 |
| • **Pancreatitis** | 5 (0.0%) | 1 (0.1%) | 0.521 |
| • **Intestinal infarction** | 4 (0.0%) | 0 (0.0%) | 0.526 |
| • **Acute abdomen bowel obstruction** | 21 (0.2%) | 5 (0.5%) | 0.073 |

Supplemental Table S2. Complications in detail between non-converted and converted cases. P-value from $\chi^2$ test reported. ARDS = adult respiratory distress syndrome; ICU = intensive care unit.

| Variable | ρ | *p-value* |
|---|---|---|
| Atrial fibrillation | 0.055 | <0.001 |
| Myocardial ischemia and infarction | 0.022 | 0.018 |
| Stroke, ictus | 0.008 | 0.38 |
| Acute peripheral arterial thromboembolism | 0.008 | 0.36 |
| Cardiac arrest | 0.032 | 0.001 |
| Prolonged air leak | 0.029 | 0.002 |
| Pulmonary embolism | 0.018 | 0.053 |
| ARDS | 0.035 | <0.001 |
| Persistent pleural space | 0.003 | 0.76 |
| Pneumonia | 0.047 | <0.001 |
| Mechanical ventilation | 0.057 | <0.001 |
| Middle lobe torsion | 0.001 | 0.92 |
| Atelectasis | 0.042 | <0.001 |
| Sputum retention | 0.036 | <0.001 |
| Haemothorax | 0.015 | 0.10 |
| Bronchopleural fistula | 0.024 | 0.01 |
| Chylothorax | -0.008 | 0.39 |
| Phrenic nerve injury/palsy | 0.004 | 0.71 |
| Recurrent laryngeal nerve palsy/dysphonia | 0.023 | 0.013 |
| Blood transfusion | 0.099 | <0.001 |
| Acute renal failure | 0.039 | <0.001 |
| Urinary tract infection | -0.008 | 0.39 |
| Dialysis | 0.002 | 0.84 |
| Diarrhoea | 0.024 | 0.009 |
| Pancreatitis | 0.006 | 0.52 |
| Intestinal infarction | -0.006 | 0.53 |
| Acute abdomen bowel obstruction | 0.017 | 0.073 |
| Prolonged mechanical ventilation | 0.031 | 0.001 |
| Postoperative ICU | 0.038 | <0.001 |

Supplemental Table S3. Spearman correlation test between conversion and complications in detail.

ARDS = adult respiratory distress syndrome; ICU = intensive care unit.

| | Logistic regression | | | | | | | | Tobit regression | | | |
|---|---|---|---|---|---|---|---|---|---|---|---|---|
| **Models** | (1) | (2) | (3) | (4) | (5) | (6) | (7) | (8) | (9) | (10) | (11) | (12) |
| | Conversion to thoracotomy | | | | ≥1 postoperative complication | | | | EuroQoL-5D score at discharge | | | |
| **Observations** | 6,363 | 4,341 | 4,341 | 4,164 | 6,363 | 4,341 | 4,341 | 4,340 | 6,348 | 4,330 | 4,330 | 4,330 |
| **>50 VATS lobectomies** | 0.78*** | 0.84* | 0.84 | 0.92 | 0.89* | 0.94 | 0.94 | 1.12 | 0.048*** | 0.057*** | 0.057* | 0.031** |
| | (0.07) | (0.088) | (0.17) | (0.13) | (0.053) | (0.066) | (0.11) | (0.10) | (0.009) | (0.011) | (0.034) | (0.012) |
| **Age** | | 1.00 | 1.00 | 0.99 | | 1.02*** | 1.02*** | 1.02*** | | -0.002*** | -0.002** | -0.002*** |
| | | (0.006) | (0.006) | (0.006) | | (0.004) | (0.005) | (0.005) | | (0.001) | (0.001) | (0.001) |
| **Female gender** | | 0.90 | 0.90 | 0.90 | | 0.80*** | 0.80** | 0.77*** | | -0.044*** | -0.044*** | -0.048*** |
| | | (0.11) | (0.13) | (0.12) | | (0.066) | (0.07) | (0.067) | | (0.013) | (0.015) | (0.011) |
| **BMI** | | 1.00 | 1.00 | 1.00 | | 1.00 | 1.00 | 1.000 | | 0.001*** | 0.001*** | 0.000*** |
| | | (0.001) | (0.002) | (0.001) | | (0.001) | (0.002) | (0.001) | | (0.000) | (0.000) | (0.000) |
| **Packages per year** | | 1.00** | 1.00** | 1.00** | | 1.00 | 1.00 | 1.000 | | -0.000** | -0.000*** | -0.000** |
| | | (0.001) | (0.001) | (0.001) | | (0.000) | (0.000) | (0.001) | | (0.000) | (0.000) | (0.000) |
| **Preoperative neoadjuvant** | | 1.38 | 1.38 | 1.76** | | 1.01 | 1.01 | 0.94 | | 0.006 | 0.006 | -0.008 |
| | | (0.33) | (0.30) | (0.45) | | (0.19) | (0.21) | (0.18) | | (0.03) | (0.026) | (0.027) |
| **FEV1 %** | | 0.99*** | 0.99** | 0.99** | | 0.99*** | 0.99*** | 0.99*** | | 0.000 | 0.000 | 0.001*** |
| | | (0.003) | (0.003) | (0.003) | | (0.002) | (0.002) | (0.002) | | (0.000) | (0.000) | (0.000) |
| **DLCO %** | | 1.00 | 1.00 | 1.00 | | 0.99*** | 0.99** | 0.99*** | | 0.001*** | 0.001** | 0.002*** |

| | | | | | | | | | | | | |
|---|---|---|---|---|---|---|---|---|---|---|---|---|
| | | (0.003) | (0.004) | (0.003) | | (0.002) | (0.003) | (0.002) | | (0.000) | (0.001) | (0.000) |
| **Right lung** | | 0.93 | 0.93 | 0.91 | | 1.04 | 1.04 | 1.04 | | -0.006 | -0.006 | -0.008 |
| | | (0.097) | (0.12) | (0.099) | | (0.073) | (0.062) | (0.076) | | (0.011) | (0.009) | (0.010) |
| **Metastasis** | | 0.64 | 0.64 | 0.54 | | 0.61 | 0.61 | 0.49* | | -0.071 | -0.071 | -0.061 |
| | | (0.34) | (0.34) | (0.30) | | (0.21) | (0.21) | (0.18) | | (0.049) | (0.073) | (0.045) |
| **Previous** | | 0.93 | 0.93 | 0.93 | | 1.07 | 1.07 | 1.02 | | 0.000 | 0.000 | 0.000 |
| **cancer-related** | | (0.11) | (0.11) | (0.12) | | (0.082) | (0.086) | (0.083) | | (0.012) | (0.019) | (0.011) |
| **surgery** | | | | | | | | | | | | |
| **Cardiac disease** | | 0.93 | 0.93 | 0.95 | | 1.32*** | 1.32** | 1.42*** | | -0.017 | -0.017 | -0.047*** |
| | | (0.14) | (0.15) | (0.15) | | (0.12) | (0.19) | (0.14) | | (0.016) | (0.021) | (0.014) |
| **COPD** | | 1.18 | 1.18 | 1.20 | | 1.50*** | 1.50*** | 1.47*** | | -0.050*** | -0.050 | -0.013 |
| | | (0.14) | (0.17) | (0.16) | | (0.11) | (0.20) | (0.13) | | (0.012) | (0.031) | (0.012) |
| **Diabetes** | | 0.87 | 0.87 | 0.90 | | 1.05 | 1.05 | 1.04 | | -0.022 | -0.022* | -0.026* |
| | | (0.13) | (0.13) | (0.14) | | (0.099) | (0.11) | (0.10) | | (0.015) | (0.013) | (0.014) |
| **Connective** | | 0.88 | 0.88 | 0.99 | | 1.11 | 1.11 | 0.93 | | -0.004 | -0.004 | 0.020 |
| **tissue disease** | | (0.32) | (0.30) | (0.37) | | (0.25) | (0.23) | (0.22) | | (0.035) | (0.047) | (0.032) |
| **Chronic kidney** | | 1.21 | 1.21 | 1.10 | | 2.40*** | 2.40*** | 2.17*** | | -0.12*** | -0.12*** | -0.053** |
| **disease** | | (0.32) | (0.32) | (0.31) | | (0.41) | (0.41) | (0.40) | | (0.028) | (0.038) | (0.025) |
| **Upper lobe** | | 0.95 | 0.95 | 0.98 | | 1.20*** | 1.20*** | 1.25*** | | -0.033*** | -0.033*** | -0.023** |
| | | (0.099) | (0.11) | (0.11) | | (0.084) | (0.078) | (0.092) | | (0.011) | (0.012) | (0.010) |

| | | | | | | | | | | | | |
|---|---|---|---|---|---|---|---|---|---|---|---|---|
| **Clinical nodal involvement** | | 1.42** | 1.42** | 1.71*** | | 1.28** | 1.28** | 1.22* | | -0.026 | -0.026 | -0.014 |
| | | (0.21) | (0.24) | (0.27) | | (0.14) | (0.16) | (0.14) | | (0.017) | (0.021) | (0.016) |
| **Clinical stage I** | | 0.62*** | 0.624*** | 0.57*** | | 0.82*** | 0.82*** | 0.85** | | 0.022* | 0.022 | 0.022** |
| | | (0.067) | (0.067) | (0.065) | | (0.060) | (0.053) | (0.066) | | (0.012) | (0.018) | (0.011) |
| **Healthcare centre dummy** | NO | NO | NO | YES | NO | NO | NO | YES | NO | NO | NO | YES |
| **Pseudo R2** | 0.002 | 0.025 | 0.025 | 0.099 | 0.001 | 0.042 | 0.042 | 0.11 | 0.004 | 0.035 | 0.035 | 0.308 |

*** p<0.01, ** p<0.05, * p<0.1

Supplemental Table S4. Logistic (1-8) and Tobit (9-12) regressions. All models feature standard errors except for models 3, 7, and 11, where standard errors are corrected for cluster correlation at the healthcare centre level.
